# Automated detection of IVC filters on radiographs with deep convolutional neural networks

**DOI:** 10.1007/s00261-022-03734-8

**Published:** 2022-11-12

**Authors:** John Mongan, Marc D. Kohli, Roozbeh Houshyar, Peter D. Chang, Justin Glavis-Bloom, Andrew G. Taylor

**Affiliations:** 1 Department of Radiology and Biomedical Imaging, Center for Intelligent Imaging, University of California San Francisco, 505 Parnassus Avenue, San Francisco, CA 94143-0628 USA; 2 Department of Radiological Sciences, Center for Artificial Intelligence in Diagnostic Medicine, University of California Irvine, Irvine, USA

**Keywords:** Inferior vena cava filter, Deep learning, Screening, Artificial intelligence

## Abstract

**Purpose:**

To create an algorithm able to accurately detect IVC filters on radiographs without human assistance, capable of being used to screen radiographs to identify patients needing IVC filter retrieval.

**Methods:**

A primary dataset of 5225 images, 30% of which included IVC filters, was assembled and annotated. 85% of the data was used to train a Cascade R-CNN (Region Based Convolutional Neural Network) object detection network incorporating a pre-trained ResNet-50 backbone. The remaining 15% of the data, independently annotated by three radiologists, was used as a test set to assess performance. The algorithm was also assessed on an independently constructed 1424-image dataset, drawn from a different institution than the primary dataset.

**Results:**

On the primary test set, the algorithm achieved a sensitivity of 96.2% (95% CI 92.7–98.1%) and a specificity of 98.9% (95% CI 97.4–99.5%). Results were similar on the external test set: sensitivity 97.9% (95% CI 96.2–98.9%), specificity 99.6 (95% CI 98.9–99.9%).

**Conclusion:**

Fully automated detection of IVC filters on radiographs with high sensitivity and excellent specificity required for an automated screening system can be achieved using object detection neural networks. Further work will develop a system for identifying patients for IVC filter retrieval based on this algorithm.

**Graphical abstract:**

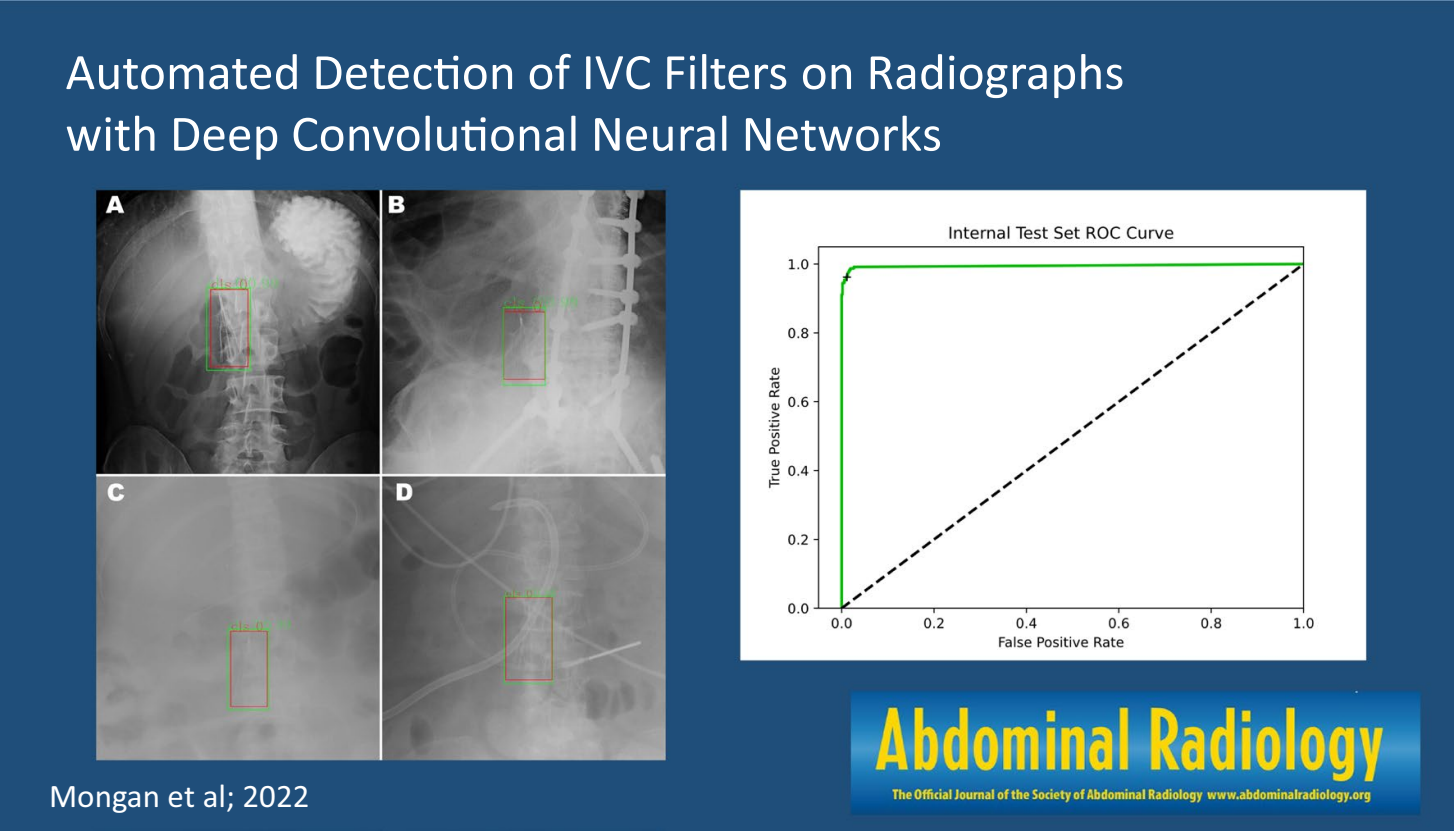

**Supplementary Information:**

The online version contains supplementary material available at 10.1007/s00261-022-03734-8.

## Introduction

Inferior vena cava (IVC) filters are used to prevent development of potentially life-threatening pulmonary embolism in patients with deep venous thrombosis (DVT), most often when patients have an absolute or relative contraindication to treatment with anticoagulation. IVC filters are protective against pulmonary embolus in the acute setting, but in some cases have demonstrated an increased risk of recurrent DVT or caval thrombosis in the long term [1, 2].

Although the majority of filters now placed are retrievable by design, filter retrieval rates are quite variable and overall fairly low in the absence of a dedicated strategy for patient follow-up [3]. Several institutions have significantly improved their retrieval rates by implementing protocols intended to reduce the number of patients being lost to follow-up [4, 5]. Further refinements of these strategies have resulted in partially automated, informatics-based approaches based on tracking patients from the time of filter placement using data from the health system’s Radiology Information System (RIS) [6]. However, by design these approaches can not identify patients who have had IVC filters placed at other institutions. This is particularly problematic because many IVC filters are placed in the setting of trauma [7], and patients may not continue with their long-term care at the trauma center where they were initially treated. An image-based rather than RIS-based approach to identifying patients who have IVC filters could help to address the challenges of tracking IVC filters in a fragmented healthcare environment where patients seek care at multiple institutions. An image-based approach could identify patients who have IVC filters placed at the medical center (from intra-procedural imaging) as well as patients who have IVC filters placed elsewhere, when those patients have abdominal imaging. An approach that identifies IVC filters directly from images rather than from radiology reports may be expected to be more transferable across institutions, as the way in which IVC filters are described in reports may vary widely, but the appearance of the filters on imaging is consistent. A successful image-based approach would require an algorithm capable of detecting IVC filters on medical imaging with high sensitivity and specificity.

Machine learning is finding widespread application in medical image analysis [8–10] and is well-suited to the task of object detection and localization. Our objective was to create an algorithm trained to identify and localize IVC filters visualized on abdominal radiographs. Further, our aim was to evaluate the generalizability of the algorithm by evaluating it at a separate institution using an independent dataset created from images obtained at that site. This algorithm is the keystone for development of a more automated process for identifying patients with IVC filters who may benefit from consultation for retrieval.

## Methods

This HIPAA-compliant retrospective study was approved by the institutional review boards of both participating institutions, which are academic, tertiary care centers; there were no external funding sources. The key points of the methods are described here; complete methods sufficient for reproducing the work are detailed in the online supplemental methods.

Candidate images from both inpatient and outpatient settings for the primary dataset were identified in our report database using mPower search software (Nuance Inc., Burlington, MA). Two searches were performed, one designed to identify abdominal radiographs where IVC filters were mentioned in the report (presumed positives) and a second designed to simply identify abdominal radiographs (presumed negative controls). Search terms and date ranges were chosen to create a dataset that would include nearly all of the images with IVC filters in our clinical archive. Corresponding images were extracted from the PACS archive.

DICOM images were annotated using the MD.ai annotation platform (MD.ai, New York, New York). Annotation of the complete dataset was performed by an attending interventional radiologist author with 13 years experience. The test partition was also annotated by two attending abdominal radiologist authors with 11 and 16 years experience, respectively. For studies with more than one image, only one representative image, selected by the interventional radiology author, was annotated. All images used were frontal images. For each annotated image, annotators either drew a bounding box around the IVC filter or marked the image as “no filter.” For the multiply annotated test set, final annotations were determined based on the majority annotation. Final bounding boxes were constructed using the mean center location and mean width and height of each annotator’s bounding boxes. The complete primary dataset was randomly divided at the patient level into training, validation and testing partitions consisting of approximately 70%, 15% and 15% of the data.

A secondary dataset, used for external validation, was constructed from images drawn from the clinical archive of a separate institution. A different instance of the same mPower search software, using the same search terms, was used to identify studies. Annotation of this dataset was performed in a custom web-based tool, but the annotation scheme was otherwise the same as for the primary dataset. All images in the secondary dataset were annotated by three radiologists: an attending abdominal radiologist with 12 years experience, an attending neuroradiologist with 7 years experience and a fourth-year radiology resident.

Annotated DICOM images were converted to JPEG format using dcmtk v3.6.2 (Offis, Oldenburg, Germany). The Cascade R-CNN [11] object detection neural network architecture using a ResNet-50 [12] backbone was employed, as implemented in MMDetection toolbox 2.4.0 [13] based on PyTorch 1.6.0 [14]. Training and inference were performed using four NVIDIA (Santa Clara, CA) RTX 2080 Ti GPUs.

Augmentation of the training partition of the dataset was performed by randomly applying transformations to the images. Applied transformations included: horizontal flip, changes in brightness and contrast, rotation in 90-degree increments, and fine rotation (1-degree increments).

Hyperparameter optimization was performed using Optuna 2.1.0 [15] to determine the best values for base learning rate, augmentation probabilities and augmentation extents. 100 iterations of optimization were performed using maximization of the area under the curve (AUC) for the receiver operator characteristic (ROC) of the model on the validation partition of the dataset as the objective function.

Using the hyperparameter values that produced the best results during hyperparameter optimization, a final model was trained on the combined training and validation partitions of the dataset. Nine additional models were trained using the same hyperparameter values but different random seeds to facilitate uncertainty estimates in the results. Final model performance was calculated based on performance on the primary internal and secondary external test sets. Confidence intervals on proportions were calculated using Chi-squared statistics using R v4.0.0 [16].

## Results

The final primary dataset consisted of 5225 annotated images, each from a separate study; 1580 of these images contained IVC filters. The dataset had a small majority of male patients and covered a broad distribution of patient ages, as shown in Table 1.

**Table 1:** Dataset characteristics

	Primary site	Secondary site
Sex (percent male)	55.2%	61.8%
Age (mean ± st dev)	57.9 ± 16.8	59.3 ± 17.9
IVC filter (percent)	30.2%	40.2%

Models trained during hyperparameter optimization had ROC AUC ranging from 0.972 to 0.995 when evaluated on the validation partition of the data. Hyperparameter values for the top performing model, identified on iteration 58 are detailed in Table 2.

**Table 2:** Optimal hyperparameter values

Hyperparameter	Value
Learning rate	0.008
90-degree rotation probability	0.125
Brightness/contrast change probability	0.278
Brightness range	− 0.154 to − 0.062
Contrast range	0.031 to 0.047
Horizontal flip probability	0.032
Fine rotation range	− 17 to 17 degrees
Fine rotation probability	0.312

**Fig. 1:**
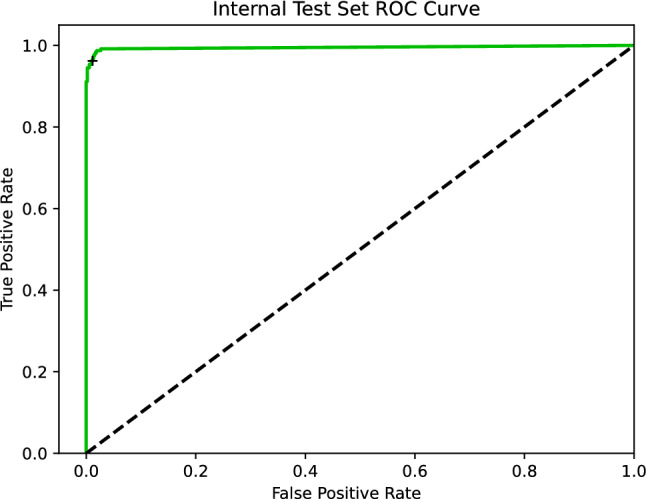
Receiver operator characteristic curve illustrating performance of the primary algorithm on the internal test set. Black cross represents the reported sensitivity and specificity

The primary model produced by this investigation, trained on the combined training and validation partitions using the optimal hyperparameter values from Table 2, had ROC AUC of 0.995 when evaluated on the internal test set (see Fig. 1). An additional nine models were trained using the same procedure as the primary model, but different random seeds; the median ROC AUC was 0.991 with an interquartile range of 0.002.

The primary model correctly recognized presence or absence of IVC filters on 746 of the 761 images in the internal test partition (see Table 3), achieving a sensitivity of 96.2% (95% CI 92.7–98.1%) and a specificity of 98.9% (95% CI 97.4–99.5%). To evaluate the generalizability of the model, the model was tested against a separately constructed and annotated dataset of 1424 images drawn from a different institution. Results running against this external test set yielded sensitivity and specificity slightly though not significantly superior to results on the primary test set: sensitivity 97.9% (95% CI 96.2–98.9%), specificity 99.6 (95% CI 98.9–99.9%), ROC AUC 0.993.

**Table 3:** Confusion matrix for primary test set. Columns represent ground truth presence or absence of IVC filter; rows represent detection or non-detection of an IVC filter by the algorithm

	Present	Absent
Detected	228	6
Not detected	9	518

The primary model evaluated an average of 15 images per second running on a single NVIDIA RTX 2080 Ti GPU.

**Fig. 2:**
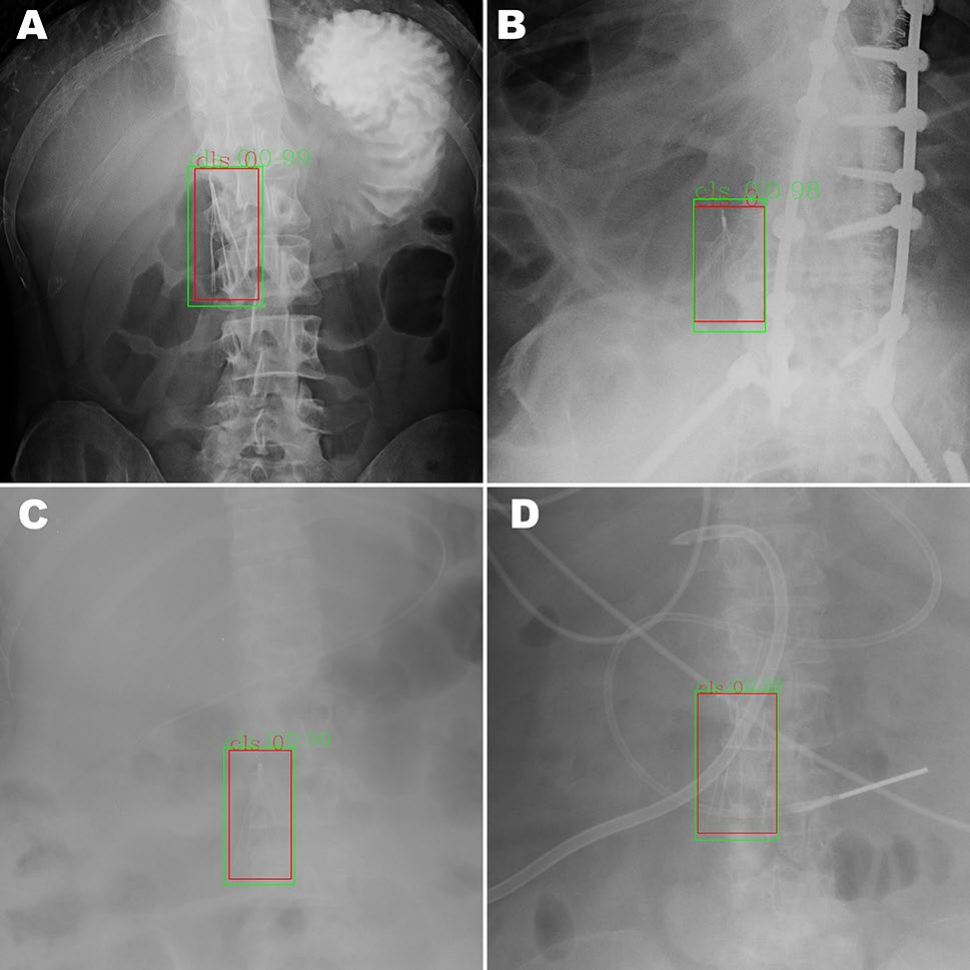
Sample of four true positive images (**A**–**D**) from the test partition of the primary dataset. Ground truth IVC filter bounding boxes annotated by radiologists are drawn in red; algorithm detections are drawn in green. Images in this figure are cropped to emphasize the region containing the IVC filter; the algorithm performed detection on full, uncropped images

**Fig. 3:**
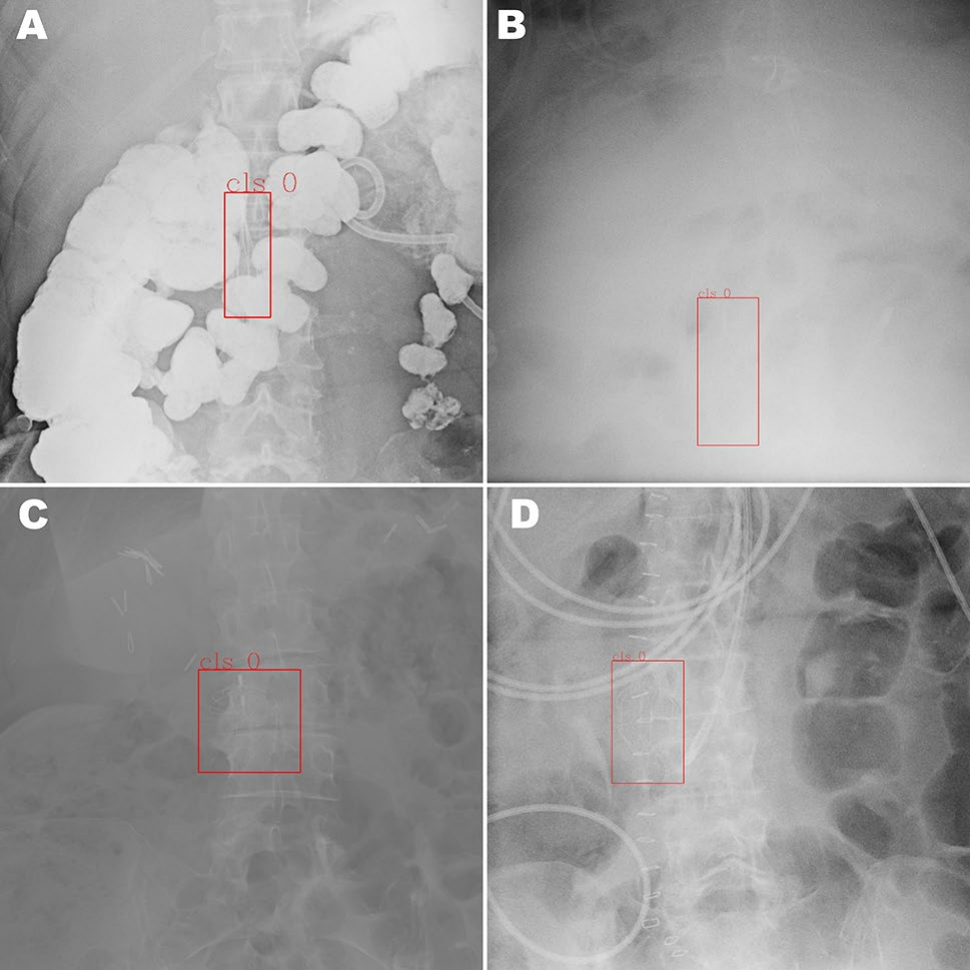
Sample of four false negative images from the test partition of the primary dataset, where the IVC filter present on the image was not detected by the algorithm. Possible factors leading to false negatives include: obscured filter (**A**), low contrast between filter and background due to technique and body habitus (**B**) and less common filter types (**C** and **D**)

**Fig. 4:**
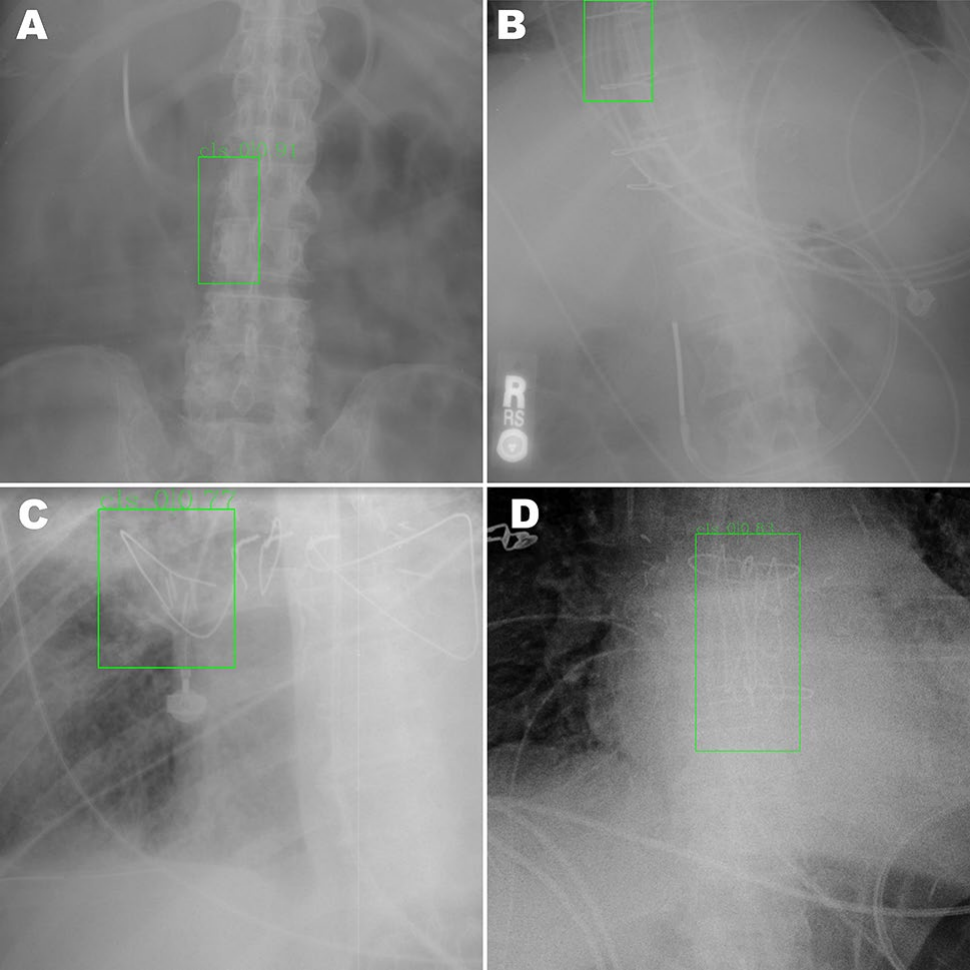
Sample of four false positive images from the test partition of the primary dataset, where the algorithm incorrectly detected non-filter structures as filters. Incorrectly detected objects include facet joints with degenerative changes (**A**), EKG leads (**B**), and sternal wires (**C** and **D**)

Figure 2 demonstrates a random sampling of correctly detected IVC filters. The model failed to detect an IVC filter in 9 images; some examples of these failures are illustrated in Fig. 3. Commonalities among the failures were heavily obscured filters, less common types of filters and low image contrast between the filter and background. In 6 images, the model incorrectly recognized non-filter objects as filters. These false positives, some of which are shown in Fig. 4, include specific examples of spinal facet joints with degenerative changes, EKG leads, and sternal wires. Note that the test partition included many additional examples of these types of findings which did not produce false positives.

## Discussion

In this investigation, we sought to create an algorithm that could detect IVC filters on radiographs, to form the foundation of an image-based system for identifying patients with filters who might be in need of retrieval. The algorithm developed here, using a deep learning object detection framework, achieves high sensitivity and specificity for detection of IVC filters on an internal test set. Perhaps more importantly, the algorithm demonstrates the same level of performance on an external test set from a separate medical center, suggesting that a system built on this algorithm would be transferable to other medical centers without loss of performance.

Other investigators have applied deep neural networks to analysis of IVC filter imaging, notably Ni et al*.*, who described an algorithm that achieves excellent results in categorizing images of filters by filter type [9]. An important distinction between the work presented here and that of Ni et al. is that the earlier work uses a *classification* network, which performs best with a tightly cropped image of an IVC filter as input, while our work uses an *object detection* network, which determines presence or absence of a filter using a full radiograph as input. This would enable our algorithm to be used for automated screening of radiographs to detect IVC filters, which the Ni et al. algorithm could not do, because it would require manual cropping around the IVC filter to match the training images and maintain adequate performance. Our algorithm does not currently classify the IVC filters it detects by type, but could be extended to include this function either by adding information on filter type to our dataset and retraining the network as a multi-class object detector, or chaining the output of our current algorithm to a classification algorithm like that in Ni et al*.* to categorize the filters detected by our algorithm. We consider this a relatively inconsequential limitation for the intended use of the algorithm in screening imaging for potentially retrievable IVC filters, as the vast majority of currently placed filters are retrievable models, and images identified as containing filters would be reviewed by an interventional radiologist prior to scheduling a retrieval attempt.

The Cascade R-CNN network architecture employed by this algorithm for object detection is a refinement of the R-CNN [17] architecture. R-CNN employs a conventional classification network (in this investigation, ResNet-50) as a backbone that classifies the contents of rectangular regions within an image; the regions are identified by a separate region proposal function. Fast R-CNN [18] improves speed by using the backbone classification network to identify features in the input image as a whole only once, rather than evaluating each of the overlapping regions separately. Faster R-CNN [19] further improves speed and accuracy by replacing the region proposal function with a neural network that operates on the features generated by the backbone classification network. The Cascade R-CNN network used here is a further extension of Faster R-CNN that introduces a multistage detector, where each successive stage refines detection using increasingly stringent thresholds to reduce false positives without negatively impacting other aspects of performance.

The algorithm created here would be of greatest use as the foundation of a system to identify and track patients with IVC filters, to ensure the filter can be retrieved when no longer required. Achieving this would require coupling our algorithm with a patient status tracking system such as that described by Juluru et al*.* [6], where our algorithm would replace the RIS query. Using an image-based algorithm would have two advantages over a RIS query. First, it would not be limited to identifying patients who had their IVC filters placed at the center where the system was being used; any patient who had abdominal radiography at the center could be identified. Second, it would enable a Digital Imaging and Communications in Medicine (DICOM) standard interface between the IVC filter tracking system and the center’s existing imaging IT infrastructure. This would likely be more portable and easier to configure than the direct query of the RIS underlying database employed by Juluru et al*.*, for which no standards exist.

Our work has several limitations and areas for improvement. It is an important building block, but it is not a clinically usable system in itself; its clinical impact cannot be directly assessed until it is incorporated into an IVC filter tracking system. Such a system would ideally identify patients based on CT as well as radiographs. This particular improvement should be achievable as a trivial extension of the algorithm described here, using scout images from CT, but this has not yet been tested. Finally, though the prospects for generalizability of this algorithm are encouraging based on the external validation results, we tested against data from only one medical center outside the center where the training data originated. There are some similarities between these two centers; in particular, both are academic medical centers. It’s possible that heterogeneity in the world of medical imaging not captured by the variation in the two centers where we tested could lead to degraded performance at other sites.

In summary, the work described here uses a general-purpose object detection network and software frameworks to achieve excellent performance in detecting IVC filters on radiographs with no manual steps. The transferability of these results on external data obtained at a separate institution is encouraging for the prospects of our future efforts to create an image-based patient tracking system to identify IVC filters in need of retrieval.

## Supplementary Information

Below is the link to the electronic supplementary material.

Supplementary file1 (DOCX 20 kb)
