# Supplementary material for: Automated detection of IVC filters on radiographs with deep convolutional neural networks
Source: Abdom Radiol (NY). 2022 Nov 12;48(2):758–64. doi: 10.1007/s00261-022-03734-8 (PMC9902407; doi:10.1007/s00261-022-03734-8)
Supplement: Supplementary file 1 — Supplementary file1 (DOCX 20 kb) [file 261_2022_3734_MOESM1_ESM.docx]

# Online Supplemental Methods

This HIPAA-compliant retrospective study was approved by the institutional review boards of both participating institutions.

Candidate images for the primary dataset were identified in our report database using mPower search software (Nuance Inc., Burlington, MA). For images expected to contain IVC filters, the terms “KUB” AND “IVC filter” were required in a search restricted by modality (XR) and date (January 1, 2000 to October 23, 2018). Search terms and date ranges were chosen to create a dataset that would include nearly all of the images with IVC filters in our clinical archive. All results returned were incorporated into the dataset. For control images, expected to not contain IVC filters, the term “KUB” alone was used, again restricted by the same modality and date modifiers. These results were returned in random sort order, and the first 3600 studies were incorporated into the dataset, with the goal of achieving an approximately 2:1 ratio of negative to positive cases. The resulting accessions for the positive and control images were used to pull images from the PACS archive. Images were deidentified at the time of extraction using a custom web-based extraction platform that embeds RSNA MIRC CTP software (RSNA, Chicago, Illinois) for deidentification functionality.

DICOM images were annotated using the MD.ai annotation platform (MD.ai, New York, New York). Annotation of the complete dataset was performed by an attending interventional radiologist author with 13 years experience. The test partition was also annotated by two attending abdominal radiologist authors with 11 and 16 years experience, respectively. For studies with more than one image, only one representative image, selected by the interventional radiology author, was annotated. For each annotated image, annotators either drew a bounding box around the IVC filter or marked the image as “no filter.” For the multiply-annotated test set, final annotations were determined based on the majority annotation. Final bounding boxes were constructed using the mean center location and mean width and height of each annotator’s bounding boxes.

A secondary dataset, used for external validation, was constructed from images drawn from the clinical archive of a separate institution. A different instance of the same mPower search software, using the same search terms, was used to identify studies. Date ranges were January 1, 2018 to December 31, 2018 for the set expected to contain IVC filters and January 1, 2018 to April 1, 2018 for the set expected to not include filters. Annotation of this dataset was performed in a custom web-based tool, but the annotation scheme was otherwise the same as for the primary dataset. For studies with more than one image, only one representative image, selected by [redacted], was annotated. Binary masks of the annotations drawn in the tool were converted to bounding boxes by determining the minimum and maximum extents of the masks on the x- and y-axes. All images in the secondary dataset were annotated by three radiologists: an attending abdominal radiologist with 12 years experience, an attending neuroradiologist with 7 years experience and a fourth-year radiology resident. Final consensus bounding boxes were constructed from each individual’s annotations using the same method as for the primary dataset.

The complete primary dataset was randomly divided into training, validation and testing partitions at the patient level, representing 70%, 15% and 15% of the patients, respectively. The number of images in each of these partitions was expected to vary slightly from these proportions as some patients had more than one imaging study. Annotated DICOM images were converted to JPEG format using the dcmj2pnm module of dcmtk v3.6.2 (Offis, Oldenburg, Germany) with --write-jpeg --min-max-window-n --grayscale switches applied.

The Cascade R-CNN^1^ object detection neural network architecture using a ResNet-50^2^ backbone was employed, as implemented in MMDetection toolbox 2.4.0^3^ based on PyTorch 1.6.0^4^. Annotation data was exported from MD.ai in JSON format and transformed into MMDetection “middle format.” Training and inference were performed using four NVIDIA RTX 2080 Ti GPUs. The “cascade_rcnn_r50_fpn_1x_coco” configuration in MMDetection was used as a starting point. The ResNet-50 backbone was initialized using weights from pre-training with ImageNet; the first of the four stages of the backbone were frozen during training of the Cascade R-CNN network. The weights outside of the backbone were initialized with random values. This configuration specified a stochastic gradient descent optimizer with momentum of 0.9 and weight decay of 0.0001. Batch size was 8. Cross entropy was used for class loss and smooth L1 was used for bounding box loss. Training proceeded for 12 epochs; learning rate decreased stepwise by a factor of 10 after epochs 8 and 11. A linear ramp learning rate warm up was employed over the first 500 steps, starting by dividing the base learning rate by 1000. Images were resized such that the long side was no more than 1333 pixels and the short side no more than 800 pixels, preserving aspect ratio.

The base configuration was modified by changing the number of classes to be detected to 1 and adding image augmentation steps to the image processing pipeline during training in addition to the random horizontal flip specified in the base configuration. These additional augmentations were performed using Albumentations 0.4.6^5^ and included changes in brightness and contrast, rotation in 90 degree increments, and fine rotation (1 degree increments). Each augmentation was applied to each image in each epoch at random, based on a probability specified for that augmentation.

Hyperparameter optimization was performed using Optuna 2.1.0^6^ to determine the best values for base learning rate, augmentation probabilities and augmentation extents. The search space for the hyperparameter optimization was learning rate 0.001-0.1 (log uniform distribution); 90 degree rotation probability 0.05-0.20; brightness/contrast probability 0-0.5, brightness centerpoint -0.15-0.15, brightness range 0-0.3, contrast centerpoint -0.15-0.15, contrast range 0-0.5; horizontal flip probability 0-0.25; fine rotation probability 0-0.5, fine rotation range 0-30 degrees. 100 iterations of optimization were performed using the default Tree-structured Parzen Estimator sampler algorithm with default settings and maximization of the area under the curve (AUC) for the receiver operator characteristic (ROC) of the model on the validation partition of the dataset as the objective function. Algorithm detection of IVC filters was considered a true positive only if the predicted bounding box had greater than 0.5 intersection-over-union overlap with a ground truth filter annotation.

Using the hyperparameter values that produced the best results during hyperparameter optimization, a final model was trained on the combined training and validation partitions of the dataset. Nine additional models were trained using the same hyperparameter values but different random seeds to facilitate uncertainty estimates in the results. Final model performance was calculated based on performance on the internal and external test sets. For external testing, a Docker (Docker, Inc; Palo Alto, California) image that included the complete software stack, framework and trained model was prepared at the primary site; the image was transmitted to the secondary site where external test set inference was performed. Confidence intervals on proportions were calculated using chi-squared statistics using R v4.0.0^7^.

# References

1. Cai Z, Vasconcelos N. Cascade R-CNN: Delving into High Quality Object Detection. CoRR 2017;abs/1712.0.

2. He K, Zhang X, Ren S, Sun J. Deep Residual Learning for Image Recognition. CoRR 2015;abs/1512.0.

3. Chen K, Wang J, Pang J, et al. MMDetection: Open MMLab Detection Toolbox and Benchmark. arXiv Prepr arXiv190607155 2019;

4. Paszke A, Gross S, Massa F, et al. PyTorch: An Imperative Style, High-Performance Deep Learning Library. In: Wallach H, Larochelle H, Beygelzimer A, Alché-Buc F, Fox E, Garnett R, editors. Advances in Neural Information Processing Systems 32. Curran Associates, Inc.; 2019. p. 8024–35.

5. Buslaev A, Parinov A, Khvedchenya E, Iglovikov VI, Kalinin AA. Albumentations: fast and flexible image augmentations. ArXiv e-prints 2018;

6. Akiba T, Sano S, Yanase T, Ohta T, Koyama M. Optuna: A Next-generation Hyperparameter Optimization Framework. In: Proceedings of the 25rd {ACM} {SIGKDD} International Conference on Knowledge Discovery and Data Mining. 2019.

7. R Core Team. R: A language and environment for statistical computing. 2020;
